# GeneXpert MTB/RIF Version G4 for Identification of Rifampin-Resistant Tuberculosis in a Programmatic Setting

**DOI:** 10.1128/JCM.02517-13

**Published:** 2014-02

**Authors:** Muhammad Osman, John A. Simpson, Judy Caldwell, Marlein Bosman, Mark P. Nicol

**Affiliations:** a City Health Directorate, City of Cape Town, South Africa; b National Health Laboratory Services, Cape Town, South Africa; c University of Cape Town, Medical Microbiology, Cape Town, South Africa; d National Division of Medical Microbiology and Institute for Infectious Diseases and Molecular Medicine, University of Cape Town, Cape Town, South Africa

## Abstract

A recent Cochrane review estimated GeneXpert MTB/RIF specificity for rifampin resistance as 98% (95% confidence interval [CI], 97 to 99), based on results from earlier test versions. The measured positive predictive value of the new generation test from programmatic implementation in Cape Town, South Africa, was 99.5% (95% CI, 98.5 to 100), confirming excellent specificity.

## TEXT

In December 2010, the World Health Organization endorsed the use of GeneXpert MTB/RIF (Xpert; Cepheid, Sunnyvale, CA), an automated nucleic acid amplification test for detection of tuberculosis and rifampin resistance for regions with high rates of HIV-tuberculosis coinfection or multidrug-resistant tuberculosis (MDR-TB) (1). The South African National Minister of Health announced a plan for the phased rollout of Xpert testing in March 2011, and South Africa is the leading adopter of Xpert testing worldwide (2). Xpert has now largely replaced smear microscopy as the primary diagnostic test for patients with presumptive tuberculosis in South Africa.

A number of studies have identified Xpert tests giving false-positive results for resistance, with specificities of 98.3% (3) in a large multicenter study and 97.5% in a smaller study (4), using a combination of phenotypic testing and targeted gene sequencing as the reference standard. The Xpert cartridge has subsequently been modified with regard to fluidics, assay settings, PCR cycling conditions, and probe B beacon sequence and with the addition of a fluorescent tracer to reduce error rates and false rifampin resistance calls (5); however, there are no compelling data to suggest that the newer version of the cartridge has improved specificity.

We describe here the measured positive predictive value of Xpert version G4 for identification of rifampin resistance during the early programmatic implementation of Xpert in Cape Town, South Africa.

We conducted a retrospective, laboratory-based record review for all patients with rifampin-resistant tuberculosis identified by Xpert from 8 August 2011 to 31 March 2012 at the Greenpoint National Health Laboratory Services (NHLS) Laboratory, Cape Town. This laboratory, which receives specimens from primary and secondary health care facilities in Cape Town, commenced testing with Xpert in August 2011.

In the Western Cape province of South Africa, two spot specimens are submitted simultaneously to the laboratory for all patients with presumptive tuberculosis. One of these specimens is tested with Xpert. If Xpert is negative and the patient is HIV infected, the second specimen is tested by culture (Bactec MGIT; Becton, Dickinson). If the Xpert result is “positive, rifampin susceptible,” the second specimen undergoes smear microscopy for programmatic monitoring and evaluation. If the Xpert result is “positive, rifampin resistant,” the second specimen is used for confirmatory drug susceptibility testing for isoniazid and rifampin using line probe assay (LPA, MTBDRplus; Hain Lifescience, Nehren, Germany). Line probe assay testing is done directly on smear-positive sputum specimens and on the cultured isolates for smear-negative specimens.

The laboratory information system of the NHLS was searched for all Xpert-positive, rifampin-resistant specimens over the study period and for matched confirmatory specimens tested within 2 months of the initial test. Since culture-based drug susceptibility testing is not performed routinely in Cape Town, we considered the result of the line probe assay test to be the reference standard for determining the positive predictive value (PPV) of Xpert.

Given names, surnames, and other personal identifiers were removed from matched data. This study was approved by the University of Cape Town, Faculty of Health Sciences Human Research Ethics Committee. Permission was obtained from the City of Cape Town Health Directorate.

From 17 October 2011 to 31 March 2012, the NHLS Greenpoint laboratory received 22,859 specimens for Xpert testing; with Xpert G4, 4,161 specimens (18.2%; 95% CI, 17.7 to 18.7) tested positive, and Xpert identified rifampin resistance in 196/4,161 (4.7%; 95% CI, 4.1 to 5.4).

**FIG 1:**
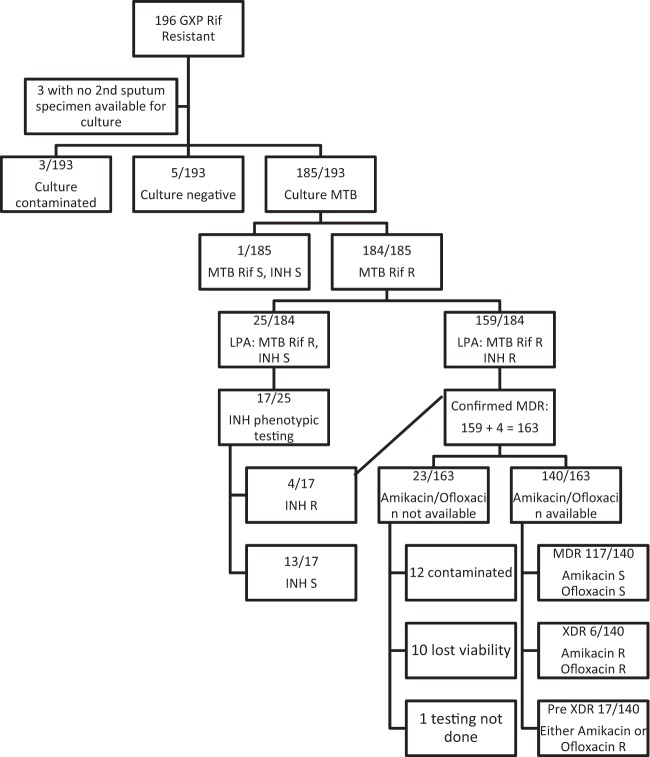
Outcome of diagnostic testing for patients with rifampin resistance identified by Xpert MTB/RIF. GXP, GeneXpert MTB/RIF; Rif, rifampin; MTB, Mycobacterium tuberculosis; INH, isoniazid; R, resistant; S, susceptible.

A second specimen was available for analysis in 193/196 (98.5%; 95% CI, 95.6 to 99.5) cases (Fig. 1). For the remaining 3 specimens, in 2 cases a second specimen was used for repeat Xpert testing due to failure of the first test; in one case, only a single specimen was submitted.

Of 193 specimens, 185 (95.9%; 95% CI, 92.0 to 97.9) were M. tuberculosis culture positive. (Of these 193, 3 [1.6%; 95% CI, 0.5 to 4.5] cultures were contaminated and 5 [2.6%; 95% CI, 1.1 to 5.9] were culture negative.) Rifampin resistance was confirmed by LPA in 184/185 (99.5%; 95% CI, 97 to 99.9); the remaining case was rifampin susceptible by the LPA, which was confirmed by phenotypic (MGIT) susceptibility testing.

Among the 184 cases with confirmed rifampin resistance on LPA, INH susceptibility testing using LPA identified INH susceptibility in 25/184 (13.6%; 95% CI, 9.4 to 19.3), 17 of the 25 had additional phenotypic INH susceptibility testing, and 4/17 (23.5%; 95% CI, 9.6 to 47.3) demonstrated INH resistance missed by LPA. One hundred sixty-three cases of MDR-TB were diagnosed (159 by LPA and 4 by phenotypic testing of INH). Susceptibility test results for amikacin and ofloxacin were available for 140/163 (85.9%; 95% CI, 79.7 to 90.4) specimens, of which 6/140 (4.3%; 95% CI, 2.0 to 9.0) were resistant to both (extensively drug resistant [XDR]).

Information on the laboratory request form indicated that of the 184 specimens with confirmed rifampin resistance, 87 (47.3%; 95% CI, 40.2 to 54.5) were from patients with no history of treatment for tuberculosis, 83 (45.1%; 95% CI, 38.1 to 52.3) were from patients previously treated for tuberculosis, and 14 (7.6%; 95% CI, 4.6 to 12.4) were from patients whose treatment history was unknown.

These results demonstrate that, in a programmatic setting in South Africa, a strategy requiring submission of two sputum samples simultaneously to the laboratory was very successful in ensuring confirmatory testing for rifampin resistance (185/196, 94.4%; 95% CI, 90.2 to 96.8), and that the positive predictive value of Xpert for rifampin resistance was 99.5% (95% CI, 98.47 to 100).

Identification of rifampin-resistant tuberculosis is an important event, both for the individual patient and from a public health perspective, triggering a cascade of interventions, including additional drug susceptibility testing, appropriate patient referral for extended and potentially toxic treatment, and contact tracing. The definitive diagnostic test should therefore have very high specificity. A recent Cochrane review (6) estimated the sensitivity and specificity of Xpert for rifampin resistance as 94% (95% CI, 87 to 97) and 98% (95% CI, 97 to 99), respectively. With these parameters and a prevalence of rifampin resistance of 5%, the positive predictive value of a rifampin-resistant result on Xpert would be 71%. The measured positive predictive value in this study of the Xpert version 4 assay is 99.5% (95% CI, 98.47 to 100). This suggests that the specificity of the new-generation Xpert assay for rifampin resistance is considerably higher than previously estimated. These results also support the decision of the South African Tuberculosis Control Programme to recommend treatment for MDR-TB on receipt of an Xpert result indicating resistance, while waiting for confirmatory testing.

We used LPA (MTBDRplus) as the reference standard for identification and confirmation of rifampin resistance. While this assay has been demonstrated to be highly sensitive and specific for rifampin resistance, it is possible that the use of phenotypic susceptibility testing may have changed our measured positive predictive value. Recent work has suggested that some forms of phenotypic susceptibility testing for rifampin may have reduced sensitivity, implying that genotypic testing may in fact be preferable (7, 8). Further, it is known that LPA has impaired sensitivity for isoniazid resistance, which may in part account for the relatively high rate of rifampin monoresistance (11.4%) in this study.

No history of previous TB treatment was recorded for almost half of the cases for which rifampin resistance was identified by Xpert, and this reinforces the importance of rapid resistance testing in patients with no history of TB and South Africa's decision to screen all patients with presumptive tuberculosis with Xpert.

Our findings may not be fully generalizable to other programmatic conditions. However, we believe that lessons learned regarding the predictive value of Xpert (G4) for rifampin resistance and the strategy of simultaneous submission of two sputum specimens may be useful for countries embarking on a similar implementation.
